# Characterization of direct oral anticoagulants use in adult hematopoietic stem cell transplant recipients

**DOI:** 10.1007/s11239-023-02902-x

**Published:** 2023-11-06

**Authors:** Claire Yang, Fatima Khan, Courtney MacDonald, Julie Guglielmo, Mimi Lo, Rebecca Young, Marisela Tan Banez, Lily Huang, Rosalyn Nguyen, Stephen Kang, Ila M. Saunders

**Affiliations:** 1 UC Davis Medical Center, 3651 Business Drive, Suite 100, Sacramento, CA 95820 USA; 2 UC San Diego Health, La Jolla, USA; 3 UC San Francisco Medical Center, San Francisco, USA; 4 UC San Diego Skaggs School of Pharmacy and Pharmaceutical Sciences, La Jolla, USA

**Keywords:** Direct oral anticoagulants, Anticoagulants, Venous Thromboembolism, Hematopoietic Stem Cell Transplantation, Hematologic Neoplasms, Pulmonary Embolism

## Abstract

Direct oral anticoagulants (DOACs) for venous thromboembolism (VTE) treatment are of interest in oncology due to ease of administration and lack of need for therapeutic monitoring compared to other anticoagulants. Data supporting their use in patients with hematologic malignancies post-hematopoietic stem cell transplant (HCT) are limited. The purpose of the study is to characterize DOAC use in HCT patients. This multicenter, retrospective cohort analysis included allogeneic and autologous HCT recipients. The primary outcome was major bleeding. Secondary outcomes included clinically relevant non-major bleeding (CRNMB)/minor bleeding and VTE recurrence. Of 126 patients, 91 (72.2%) patients received an autologous HCT, and 35 (27.8%) patients received an allo-HCT. No major bleeding occurred in either transplant recipient groups. In autologous HCT recipients, CRNMB/minor bleeding occurred in four (4.4%) patients and VTE recurrence occurred in one (1.1%) patient. For allogeneic HCT recipients, CRNMB/minor bleeding occurred in five (14.3%) patients and VTE recurrence occurred in two (5.7%) patients. For patients that experienced a CRNMB, five (100%) of the allogeneic HCT and two (50%) of the autologous HCT recipients were thrombocytopenic at the time of bleeding. Only 38.5% of patients who experienced a drug-drug interaction requiring DOAC dose adjustment received the appropriate dose adjustment. DOACs were associated with low rates of recurrent VTE and no major bleeding events, similar to published data on DOAC use in the general cancer patient population. This suggests that DOACs may be safe therapeutic options with proactive management of drug interactions and careful monitoring for bleeding events, especially in the allogeneic HCT population where minor bleeding rates were slightly higher.

## Highlights

- Direct Oral Anticoagulants (DOACs) are a reasonable anticoagulant option for hematopoietic stem cell transplant recipients and were associated with low rates of major bleeding and venous thromboembolism recurrence in this underrepresented patient population.
- VTE recurrence rate of 1.1% in the autologous HCT population was lower while VTE recurrence rate of 5.7% in the allogenic HCT population was comparable to studies in the general oncologic population.
- Allogeneic HCT recipients receiving DOACs for therapeutic anticoagulation may be at greater risk for clinically relevant non–major/minor bleeding compared to autologous HCT recipients.
- Prior to the initiation of DOACs in HCT recipients, a risk/benefit discussion regarding bleeding risks, proactive dose adjustments for concomitant drug-drug interactions, and robust patient education is imperative to optimize patient safety.
- Future studies that prospectively compare DOACs to LMWHs in a controlled setting are warranted to corroborate these findings.

## Introduction

Approximately 20% of all cases of venous thromboembolisms (VTE) occur in patients with cancer. VTE affects up to 20% of patients with cancer before death and has been reported in up to half of postmortem examinations of cancer patients [1]. Many cancer therapies, including surgery, hormonal therapy, and select chemotherapy agents, (i.e., immunomodulatory drugs) can increase the risk of VTE [1].

The 2021 American Society of Clinical Oncology guidelines provide robust recommendations regarding prophylaxis and treatment of VTE in patients with cancer, including direct oral anticoagulants (DOACs), such as apixaban, rivaroxaban and edoxaban, in addition to low molecular weight heparins (LMWHs) as first line options for VTE treatment [2]. The 2023 National Comprehensive Cancer Network (NCCN) Cancer-Associated VTE Guidelines recommend select DOACs (apixaban, edoxaban, and rivaroxaban) as category 1 recommendations for cancer-associated VTE treatment, preferred for patients without gastric or gastroesophageal lesions given increased bleeding risk. Dalteparin, a LMWH, is also listed as a category 1 recommendation preferred for patients with gastric and gastroesophageal lesions [3]. Evidence for these recommendations is largely derived from studies evaluating the use of DOACs compared with LMWHs and vitamin K antagonists in cancer populations [4–6]. Data from these trials, such as ADAM-VTE, HOKUSAI, CARAVAGGIO and SELECT-D, have suggested that bleeding and VTE recurrence rates are low compared to LMWHs, deeming DOACs to be safe therapeutic options in the general oncology population [4–7].

DOACs have increasingly been seen as attractive treatment options given the ease of administration and evidence of effectiveness in patients with cancer-associated VTE. However, the American Society of Hematology (ASH) panel suggests more information is needed on the dosing of anticoagulation for patients with hematological malignancies or undergoing hematopoietic stem cell transplantation (HCT) [1]. Patients with hematologic malignancies are severely underrepresented in DOAC landmark clinical trials, comprising only 5.7–10% of the entire study population. Even in studies such as the ADAM-VTE, HOSUKAI, CARAVAGGIO, and SELECT-D studies which specifically review DOACS in patients with malignancies, information regarding the inclusion of HCT recipients is bereft in this high acuity patient population.

Patients who undergo HCT have several risk factors for both thrombosis and bleeding. HCT is associated with an acquired hypercoagulable state, indicated by increased inflammation, coagulation factors, and von Willebrand factor [8]. Other risk factors that contribute to thrombosis risk include graft-versus-host disease (GVHD), the use of indwelling venous catheters, and use of immunomodulatory agents post-HCT in select hematologic malignancies. Alternatively, HCT recipients, specifically allogeneic recipients, are also at risk for bleeding due to prolonged and severe thrombocytopenia post-HCT, as well the potential for drug-drug interactions [8, 9]. Notably, no studies have characterized the safety and effectiveness of DOAC use in HCT recipients. This study is a multicenter, retrospective cohort analysis designed to characterize the safety and efficacy of DOACs for the treatment of VTE in patients that have undergone HCT.

## Methods

### Patients

This multicenter, retrospective, observational study included patients over the age of 18, diagnosed hematologic malignancy according to the World Health Organization criteria [10], received a hematopoietic stem cell transplant (autologous-HCT (auto-HCT) or allogeneic-HCT (allo-HCT), diagnosed with VTE 100 days prior to or 100 days post-HCT (since the risk for adverse bleeding events and recurrent VTE tend to be highest in the first 3 months after the first VTE occurrence), and received at least one month of a DOAC for treatment of their VTE. The intention for requiring at least one month of DOAC therapy was to ensure better correlates with clinical findings and accurately reflect the patient population. If an outcome event was noted, confirmation of DOAC use at the time of event was conducted. VTE types included in this study were deep venous thromboembolism (DVT) (including peripherally inserted central catheter (PICC)-associated VTE) and pulmonary embolism (PE) [10]. Patients were excluded if they had a solid tumor malignancy without hematologic malignancy, received non-FDA approved dosing of a DOAC for VTE treatment (Table 4)*,* and if they were not monitored by their provider at least monthly at any of the participating institutions during the study period. Patients in this study received their HCT from one of three participating National Cancer Institute (NCI)-designated Comprehensive Cancer Centers in California: University of California, Davis Health (UCDH) Comprehensive Cancer Center, University of California, San Diego (UCSD) Moores Cancer Center, and University of California, San Francisco (UCSF) Hellen Diller Comprehensive Cancer Center. The study period spanned approximately nine years: from July 1, 2012 to May 18, 2021 and patients were monitored for six months after date of DOAC initiation. Drug interactions were captured for patients who were on a concomitant P-glycoprotein (p-gp)/strong cytochrome P450 3A4 (CYP3A4) inhibitor (i.e., posaconazole, voriconazole, clarithromycin), as well as whether the DOAC was appropriately adjusted (Table 5) or withheld based on manufacturer recommendations when co-administered with P-gp and/or strong CYP3A4 inhibitors [11–14]. Clinical review was conducted by clinical pharmacists at each of the three participating institutions using the electronic medical record system. All clinical notes by physicians, nurses, and pharmacists documented during the study period and pertinent laboratory values were utilized to inform the outcomes studied.

### Outcome measures

The primary safety endpoint was any episode of major bleeding. Major bleeding was defined according to International Society on Thrombosis and Haemostasias (ISTH) criteria and modeled after previous studies: overt bleeding plus a hemoglobin decrease of ≥ 2 g/dL or transfusion of ≥ 2 units of packed red blood cells; bleeding in a critical site (intracranial, intraspinal/epidural, intraocular, retroperitoneal, pericardial, intraarticular, intramuscular with compartment syndrome), or fatal bleeding [15]. Secondary outcomes included any episode of clinically relevant non-major bleeding (CRNMB) or minor bleeding per ISTH criteria and VTE recurrence. Clinically relevant non-major bleeding (CRNMB) was defined as overt bleeding not meeting the criteria for major bleeding but associated with medical intervention, an unscheduled contact with the health care team, or temporary anticoagulant cessation. Minor bleeding was defined as overt bleeding not meeting criteria for major bleeding or CRNMB. All outcome measures were recorded within 6 months of the date of DOAC initiation in each patient.

### Patient demographics

Patient demographic and clinical characteristics assessed include sex, stem cell transplant type (auto-HCT or allo-HCT), stem cell source (peripheral blood, cord blood, or bone marrow), age at time of transplant, race, type of malignancy, indication for anticoagulation (VTE, PE, or line-associated VTE), platelets at baseline and at the time of the bleeding event, HAS-BLED score, and creatinine clearance (CrCl) at baseline prior to DOAC initiation. Other patient data collected at baseline include presence of drug-drug interactions at time of DOAC initiation and time from DOAC initiation to bleed. Drug metabolism interactions documented included strong CYP3A4/P-glycoprotein inducers (i.e., carbamazepine, phenobarbital, phenytoin, rifampin, or St John’s wort) and strong CYP3A4/P-glycoprotein inhibitors (defined as use of HIV protease inhibitors, posaconazole, clarithromycin, telithromycin, nefazodone, amiodarone, diltiazem, dronedarone, or verapamil) [11–14]. Select medications that may increase risk of VTE (i.e. bevacizumab, erythropoietin, estrogens, progestins, lenalidomide, pomalidomide, thalidomide, and tamoxifen) in this patient population were also assessed [17]. Concomitant antiplatelet therapy (aspirin, clopidogrel, ticagrelor, and prasugrel), which may increase bleeding risk, were also documented.

### Statistical considerations

Patient characteristics and outcome measurements were reported using descriptive statistics as frequencies and percentages.

## Results

**Fig. 1:**
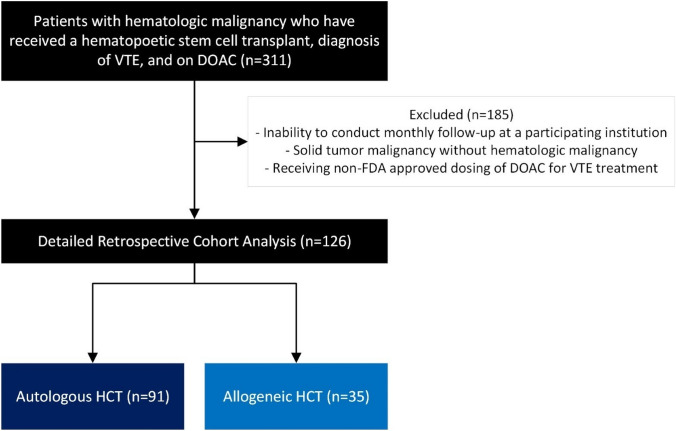
Patient Consort Diagram

A total of 311 patients were assessed for eligibility, and 126 patients were included in the final analysis (Fig. 1). The most common reason for exclusion was inadequate provider follow up. This study required that patients be seen monthly at a participating institution in order to adequately capture changes in patients’ clinical statuses, diagnoses, and laboratory assessments. The second most common reason for exclusion was diagnosis of VTE outside of the specified window specified by the study. Of those, 91 (72.2%) patients received an auto-HCT, and 35 (27.8%) patients received an allo-HCT. Most patients were male (n = 79, 62.7%), white (n = 97, 77%), and received peripheral blood as the stem cell source (91.4% of allo-HCT (n = 32)) (Table 1). The majority of patients had a DVT diagnosis (n = 106, 84.1%). Of those patients with a DVT diagnosis, 27% were PICC-associated thromboembolism. Most patients received rivaroxaban (n = 66, 52.4%). Of note, at DOAC initiation, 55 (43.7%) patients were thrombocytopenic with platelets of 50 K/mm^3^ to 150 K/mm^3^ and 9 (7.1%) patients had a platelet count less than 50 K/mm^3^. The most common malignancy types in the auto-HCT population were multiple myeloma (n = 68, 74.7%) and non-Hodgkin lymphoma (n = 16, 17.6%). The allo-HCT population predominantly consisted of patients diagnosed with acute myeloid leukemia (n = 17, 48.6%), acute lymphocytic leukemia (n = 6, 17.1%), and non-Hodgkin lymphoma (n = 6, 17.1%).

**Table 1:** Characteristics of Patients at Baseline

Characteristic	Total (N = 126)	Autologous (n = 91)	Allogeneic (n = 35)
Male sex—no. (%)	79 (62.7)	53 (58.2)	26 (74.3)
Mean Age at Time of Transplant (years)	58.9	56.8	59.8
Stem Cell Transplant Type—no. (%)	126 (100)	91 (72.2)	35 (27.8)
Stem Cell Source—no. (%)			
Peripheral blood	123 (97.6)	91 (100)	32 (91.4)
Bone marrow	3 (2.4)	0 (0.0)	3 (8.6)
Race—no. (%)			
White	97 (77.0)	73 (80.2)	24 (68.6)
African American	5 (39.7)	4 (4.4)	1 (2.9)
Asian	5 (39.7)	3 (3.3)	2 (5.7)
Hispanic	8 (6.4)	4 (4.4)	4 (11.4)
Native American or Alaskan Indian	1 (0.8)	1 (1.1)	0 (0.0)
Other	10 (7.9)	6 (6.6)	4 (11.4)
Type of Malignancy—no. (%)			
Multiple Myeloma, Amyloidosis, Plasma Cell Leukemia	68 (54.0)	68 (74.7)	0 (0.0)
Hodgkin Lymphoma	5 (4.0)	5 (5.5)	0 (0.0)
Non-Hodgkin Lymphoma	22 (14.3)	16 (17.6)	6 (17.1)
Acute Myeloid Leukemia	18 (14.3)	1 (1.1)	17 (48.6)
Acute Lymphocytic Leukemia	6 (4.8)	0 (0.0)	6 (17.1)
Myelodysplastic Syndrome	4 (3.2)	0 (0.0)	4 (11.4)
Chronic Lymphocytic Leukemia	1 (0.8)	0 (0.0)	1 (2.9)
Chronic Myeloid Leukemia	2 (1.6)	0 (0.0)	2 (5.7)
Indication for Anticoagulation—no. (%)			
Deep Venous Thromboembolism	106 (84.1)	75 (92.4)	31 (88.6)
Pulmonary Embolism	20 (15.9)	16 (17.6)	4 (11.4)
Anticoagulant—no. (%)			
Rivaroxaban	66 (52.4)	48 (45.3)	18 (51.4)
Apixaban	56 (44.4)	42 (46.2)	14 (40.0)
Edoxaban	2 (1.59)	0 (0.0)	2 (5.7)
Dabigatran	2 (1.59)	1 (2.9)	1 (2.9)
Platelet count—K/mm^3^ (Baseline)			
< 50	9 (7.1)	7 (7.7)	2 (5.7)
50–100	32 (25.4)	17 (18.7)	15 (42.9)
101–150	23 (18.3)	17 (18.7)	6 (17.1)
150–450	60 (47.6)	48 (52.7)	12 (34.3)
> 450	2 (1.6)	2. (2.2)	0 (0.0)
HAS-BLED Score—no. (%)			
0	49 (47.1)	35 (38.5)	14 (40.0)
1	42 (40.4)	29 (31.9)	13 (37.1)
2	12 (11.5)	11 (12.1)	1 (2.9)
3	1 (0.8)	1 (2.9)	0 (0.0)
Creatinine Clearance (mL/min)			
> 90	68 (54.0)	50 (55.0)	18 (51.4)
60–89	46 (36.5)	32 (35.2)	14 (40.0)
30–59	12 (9.5)	9 (9.9)	3 (8.6)
< 30	0 (0.0)	0 (0.0)	0 (0.0)
Drug Drug Interactions—Medications that may Increase Clot Risk^Ω^ —no. (%)	21 (16.7)	19 (20.9)	2 (5.7)
Drug Drug Interactions- Antiplatelets Present^α^—no. (%)	8 (6.3)	8 (8.8)	0 (0.0)
Drug Drug Interactions Drug Metabolism Interaction with- no. (%)			
Strong CYP3A4/Pgp inhibitor	13 (10.3)	1 (1.1)	12 (34.3)
Was DOAC dose appropriately adjusted? (n = 13)			
Yes	5 (38.5)	1 (20.0)	4 (34.3)
No	8 (61.5)	0 (0.0)	8 (100.0)
DOAC Status 6 Months Post Initiation – no. (%)			
Continued	79 (62.7)	62 (68.1)	17 (48.6)
Discontinued	42 (33.3)	25 (27.5)	17 (48.6)
Temporarily Held	0 (0.0)	0 (0.0)	0 (0.0)
Switched to a different DOAC	5 (4.0)	4 (4.4)	1 (2.9)

^Ω^Bevacizumab, Erythropoietin, Estrogens, Lenalidomide, Pomalidomide, Progestins, Tamoxifen, Thalidomide

^α^Aspirin, Clopidogrel, Ticagrelor, Prasugrel

Primary Outcome: Major bleeding as defined by ISTH criteria did not occur in either the auto–HCT or allo-HCT populations. Secondary Outcomes: CRNMB or minor bleeding occurred in three (3.3%) patients in the auto-HCT and five (14.3%) patients in the allo-HCT population (Table 2). Of those, one (33%) of the auto-HCT and five (100%) of the allo-HCT recipients were thrombocytopenic at the time of bleeding. VTE-recurrence occurred in one (1.1%) patient in the auto-HCT and two patients (5.7%) in the allo-HCT population (Table 2).

**Table 2:** Characteristics of Primary and Secondary Outcomes

Patient Number	DOAC^Δ^	On DOAC at time of Bleed	Time to Bleed from DOAC start (Days)	Platelets at the time of Bleed (K/mm^3^)	Drug Drug Interactions—Antiplatelets Present at Time of Event	Drug Drug Interactions—Drug Metabolism Interaction with DOAC at Time of Event
Clinically Relevant Nonmajor Bleeding/Minor Bleeding						
Autologous- HCT						
1	Apixaban	Yes	83	163	No	No
2	Rivaroxaban	Yes	62	206	No	No
3	Rivaroxaban	Yes	159	125	No	No
Allogeneic-HCT						
1	Apixaban	Yes	4	116	No	Yes, posaconazole
2	Apixaban	Yes	166	15	No	Yes, posaconazole
3	Rivaroxaban	Yes	6	101	No	No
4	Rivaroxaban	Yes	31	139	No	No
5	Rivaroxaban	Yes	111	119	No	No

Patient Number	DOAC^Δ^	Initial Diagnosis	Recurrent Diagnosis	Time to recurrence from transplant (days)	Time to recurrence from DOAC start (days)	Drug interaction present^Σ^
Recurrent VTE						
- Autologous						
1	Rivaroxaban	PICC-associated	PICC-associated	20	14	Yes, lenalidomide
- Allogeneic						
2	Apixaban	DVT	PE	150	123	Yes, posaconazole
3	Rivaroxaban	PICC-associated	PICC-associated	90	32	No

^Δ^ FDA-approved dosing strategies employed for the treatment of venous thromboembolism per individual DOAC package inserts (unless stated otherwise)

^Σ^ Drug metabolism or drug increasing risk of blood clots present at time of VTE recurrence

Thirteen (10.3%) patients had a concomitant p-gp/strong 3A4 inhibitor while on a DOAC. The concomitant drug interaction for all of these patients was with posaconazole. Five of these patients (38.5%) had their DOAC dose adjusted appropriately and of these five, one of them still experienced a minor bleed. This patient was an allo-HCT recipient and the minor bleeding criteria met was hematuria. At the time of the bleed, the patient was diagnosed with human polyoma virus 1 (BK virus) which when coupled with anticoagulation likely contributed to CRNMB despite appropriate dose adjustment of apixaban and platelets of 116 K/ mm^3^. Notably, this patient was not receiving concomitant antiplatelet therapy; their DOAC was held for one week following the report of hematuria and the DOAC was subsequently continued with no further bleeding episodes throughout the study evaluation period. Of the eight patients (61.5%) who did not have their DOAC dose adjusted, one patient experienced a minor bleed. This patient was an allo-HCT recipient and the minor bleeding criteria met was epistaxis. Their platelet count at the time of their minor bleed event was 15 K/mm^3^ with no concomitant antiplatelet therapy reported; the DOAC was not held before, during, or after the epistaxis event. Two of the patients who experienced a bleed were concurrently on steroid therapy (i.e. prednisone, prednisolone or dexamethasone) at the time prior to or time during the event. However, none of the associated bleeds were gastrointestinal in nature. Moreover, all patients who received an allo-HCT and experienced an outcome event were assessed for incidence of transplant-related complications including, GVHD, thrombotic microangiography (TMA), and sinusoidal obstructive syndrome (SOS). One patient was diagnosed with grade II GVHD while none of the patients with an outcome event were diagnosed with TMA or SOS.

## Discussion

The primary aim of the current study was to characterize DOAC use in patients with hematologic malignancies post-HCT. With all VTEs, the bleeding risk and prevention of recurrent VTE can be difficult to balance, especially in the post-HCT setting. Given that most of the patients in this study were on either apixaban or rivaroxaban, we compared the results of our study to the SELECT-D (rivaroxaban), CARAVAGGIO (apixaban), and ADAM VTE (apixaban) trials (Table 3). Patients with hematologic malignancies were underrepresented in the SELECT-D, CARAVAGGIO, and ADAM VTE trials, representing only 6.9%, 5.7% and 8.7% of all patients, respectively. Major bleeding occurred in none of the patients in this study as compared to 5.4%, 3.8%, and 0% in the SELECT-D, CARAVAGGIO, and ADAM VTE trials, respectively. CRNMB/minor bleeding rates in the auto-HCT group were 3.3% which is slightly less than published values in the general cancer population. However, CRNMB/minor bleeding rates were noticeably higher in the allo-HCT group. This difference was anticipated due to greater immunosuppression in the allo-HCT group resulting in platelet level fluctuations, thrombocytopenia and greater risk for infections and GVHD [16]. The higher risk for thrombocytopenia was substantiated by the results from this study, as the average platelet count in patients who experienced a CRNMB/minor bleed in the allo-HCT recipients was noticeably lower at the time of event (98 K/mm^3 compared to 165 K/mm^3^ in auto-HCT recipients). VTE recurrence rate of 1.1% in the auto-HCT population was lower compared to previous studies in the general oncologic population which reported VTE recurrence rates 4% to 7.9% while VTE recurrence rate of 5.7% in the allo-HCT population was comparable [4–7].

**Table 3:** Comparison of bleeding and VTE recurrence to previous trials

Current Studies (Date)	DOAC(s) Studied	Sample Size on DOAC (n)	Minimum Treatment Duration (months)	Hematologic Malignancies on DOAC (%)^δ^	Major Bleed (%)	CRNMB/Minor Bleed (%)	VTE Recurrence (%)
SELECT-D (2018)	Rivaroxaban	202	6	6.9	5.4	12.3	4.0
CARAVAGGIO (2018)	Apixaban	576	6	5.7	3.8	10.2	5.6
ADAM VTE (2020)	Apixaban	145	6	8.7	0.0	6.2	Not studied
Hokusai-VTE (2018)	Edoxaban	522	6	Not reported	6.9	14.6	7.9
UC Collaborative—Allogeneic	Apixaban Rivaroxaban Edoxaban Dabigatran	35	1	100	0.0	14.3	5.7
UC Collaborative—Autologous		91		100	0.0	3.3	1.1

^δ^Did not count as hematologic malignancy if cancer type was characterized as 'other' or 'unknown'

The primary drug-drug interaction seen in this study was with DOAC and posaconazole. Posaconazole is an antifungal agent that is used for prophylaxis as yeasts and molds can cause serious invasive fungal infections in HCT recipients. However, posaconazole does have a strong inhibitory effect on hepatic CYP3A4 and is both a P-gp substrate and inhibitor. When utilized concurrently with select DOACs, DOAC serum plasma levels are expected to increase leading to recommendations for DOAC dose decrease to mitigate the increased risk of bleeding (Table 5)*.* This study demonstrated that less than half (only 38.5%) of patients who experienced this interaction had their DOAC dose adjusted appropriately highlighting the need to increase meticulous screening for concomitant drug-drug interaction to adequately decrease bleed risk in the HCT patient population.

To our knowledge, this is the first study to assess the utilization of DOACs in the hematopoietic stem cell transplant population. This study provides value by differentiating between allo-HCT and auto-HCT, highlighting clinical differences amongst the populations. Additionally, our population was sizeable, at 126 patients and represented multiple sites. Patients in this study received their transplant at one of three large, NCI-designated comprehensive cancer centers associated with three major academic medical centers in California over nine years.

Limitations of this study include the relatively short treatment duration and retrospective study design. Previous prospective trials required the DOAC minimum treatment duration to be 6 months. For this study, the minimum treatment duration was 1 month. Patients were monitored for the event rate through six months after initiation of the DOAC. Due to the short duration of follow-up, the potential for the development of long-term adverse effects cannot be ruled out and the retrospective design limits the involvement of a direct comparison group. Thus, superiority or noninferiority to alternative agents such as LMWH was not assessed. In addition, use of GVHD prophylaxis and evidence of TMA were not assessed universally in the allo-HCT population [17].

Several guiding organizations have suggested that if drug interactions are present, the use of DOACs should be carefully assessed, and in some cases, avoided. In cases where potential drug-drug interactions with DOACs are not able to be avoided, LMWH is preferred [16]. Each of the DOACs is a substrate for P-gp, an efflux transporter located in the gut mucosa, and therefore, all DOACs are susceptible to drugs that induce or inhibit P-gp.

Eight patients out of the 13 that had a relevant drug interaction (61.5%) did not have their DOAC dose appropriately adjusted or withheld per individual DOAC package insert recommendations. Five of these 8 patients continued rivaroxaban at standard VTE treatment dosing, despite the existing recommendation to discontinue treatment with rivaroxaban due to this drug interaction. Notably, none of these patients experienced a major or CRNMB/minor bleed. Three of the eight patients continued the normal dose of apixaban at 5 mg orally twice daily. One of the 5 patients that did not have their DOAC dose adjusted appropriately to 2.5 mg orally twice daily, experienced a minor bleed 5 months into DOAC treatment. The minor bleed experienced was a single documented episode of epistaxis accompanied by thrombocytopenia (platelet count of 15 K/mm^3^), for which apixaban was subsequently discontinued. Thus, it may be feasible to use DOACs in the setting of such drug interactions, with careful consideration for dose-reduction of the DOAC.

## Conclusion

This multicenter retrospective study suggests that the use of DOACs in the HCT population may be safe and effective, given similar efficacy and safety when compared to published literature in the general oncologic population. Due to the higher frequency of CRNMB/minor bleeding in allo-HCT recipients receiving DOACs, a risk/benefit discussion prior to DOAC initiation, robust patient education, and careful monitoring of laboratory values and clinical signs of bleeding is strongly warranted. Finally, drug interactions must also be carefully and continuously assessed, ensuring that concomitant p-gp/strong CYP3A4 inhibitors result in appropriate dose adjustment or discontinuation of the DOAC for an alternative anticoagulant. Future studies that prospectively compare DOACs to LMWHs in a controlled setting are warranted to corroborate these findings.

## Appendix

See Tables 4, 5

**Table 4:** FDA Approved dosing for DOACs for VTE Treatment

DOAC	Recommended Dosing per DOAC Manufacturer
Apixaban (Eliquis) [11]	10 mg twice daily for 7 days followed by 5 mg twice daily No dosage adjustment is recommended by the manufacturer for any degree of reduced kidney function
Rivaroxaban (Xarelto) [12]	15 mg twice daily with food for 21 days followed by 20 mg once daily with food CrCl ≥ 30 mL/minute: no dosage adjustment necessary. Observe closely and promptly evaluate any signs or symptoms of blood loss in patients with CrCl 15 to < 30 mL/min. Avoid the use in patients with CrCl < 15 mL/min
Edoxaban (Savaysa, Lixiana) [13]	The recommended dose is 60 mg taken orally once daily following 5 to 10 days of initial therapy with a parenteral anticoagulant The recommended dose is 30 mg once daily in patients with CrCL 15 to 50 mL/min, patients who weigh less than or equal to 60 kg, or patients who are taking certain concomitant P-gp inhibitor medications

**Table 5:** Appropriate DOAC Adjustments with concomitant P-gyp/strong CYP3A4 inhibitors

DOAC	Recommended Dosing per DOAC Manufacturer
Apixaban (Eliquis) [11]	For patients receiving doses of 5 mg or 10 mg twice daily, reduce the dose by 50% when coadministered with drugs that are combined P-gp and strong CYP3A4 inhibitors (e.g., ketoconazole, itraconazole, or ritonavir). In patients already taking 2.5 mg twice daily, avoid coadministration with combined P-gp and strong CYP3A4 inhibitors
Rivaroxaban (Xarelto) [12]	Avoid concomitant use with known combined P-gp and strong CYP3A inhibitors
Edoxaban (Savaysa, Lixiana) [13]	The recommended dose is 30 mg once daily in patients who are taking certain concomitant P-gp inhibitor medications (i.e. rifampin). No dosage adjustment necessary with strong CYP3A4 inhibitors
